# A Comparison between Platelet-Rich Plasma (PRP) and Hyaluronate Acid on the Healing of Cartilage Defects

**DOI:** 10.1371/journal.pone.0097293

**Published:** 2014-05-12

**Authors:** Ji Liu, Wenqi Song, Ting Yuan, Zhengliang Xu, Weitao Jia, Changqing Zhang

**Affiliations:** Department of Orthopaedic Surgery, Shanghai Sixth People's Hospital, Shanghai Jiao Tong University School of Medicine, Shanghai, China; University of Pittsburgh, United States of America

## Abstract

Platelet-rich plasma (PRP) has offered great promise for the treatment of cartilage degradation, and has been proved to have positive effects on the restoration of cartilage lesions. But no comparative work has been done between PRP and hyaluronate acid (HA) concerning their restoring effect on cartilage defect, especially by means of animal experiments and histologic assessments. The purpose of the study was to compare the therapeutic effects of P-PRP and HA on osteoarthritis in rabbit knees. Thirty rabbits were used to establish the animal models by creating a cartilage defect of 5 mm in diameter on the condyles of the femurs, and were randomly divided into three groups: the P-PRP group, HA group and the control group. Then each group was treated with P-PRP, HA or saline solution, respectively. Six and twelve weeks later the rabbits were sacrificed and the samples were collected. The platelet number, the concentrations of growth factors of P-PRP and whole blood, and the IL-1β concentration in the joint fluid were investigated, and the histological assessment of the cartilage were performed according to Mankin's scoring system. Micro-CT was also used to evaluate the restoration of subchondral bone. The platelet concentration in P-PRP is 6.8 fold of that in the whole blood. The IL-1β level in the P-PRP group was lower than in the HA group (p<0.01) and in the control group (p<0.01). The restoration of the defected cartilage as well as the subchondral bone was better in the P-PRP group than in the HA group or the control group (P<0.05). Our data showed that P-PRP is better than HA in promoting the restoration of the cartilage and alleviating the arthritis caused by cartilage damage.

## Introduction

Due to its poor blood supply and self-renewal capacity, the normal structure and function of cartilage are difficult to restore when it's injured or degenerated. The pathological changes involve the degeneration or inflammatory reaction of the cartilage, the subchondral bone and synovium, even the formation of osteophytes. The conventional treatments for cartilage degeneration or osteoarthritis (OA) include intra-articular injection of lubricant or anesthetic medication or arthroplastic surgery. Hyaluronic acid (HA) is a kind of acidic mucopolysaccharide, which has been used as a conventional lubricant for degenerated joints for decades [1]. It can lower the friction between the articular surfaces, and alleviate the joint pain of the patients, but its effect is not longstanding and in some occasions may cause allergic or inflammatory reaction. Platelet-rich plasma (PRP) is a concentrate of autologous platelets, and platelet concentrates techniques could be classified in 4 families based on their fibrin architecture and leukocyte content: pure platelet-rich plasma (P-PRP) and leukocyte- and platelet-rich plasma (L-PRP) are liquid platelet suspensions; P-PRP gel and L-PRP gel are polymerized fibrin gel, when P-PRP and L-PRP are activated, respectively [2]. Pure platelet-rich fibrin (P-PRF) and leukocyte- and platelet-rich fibrin (L-PRF) are solid fibrin materials, and cannot be injected as liquid solution. PRP has been widely used to promote the recovery of the soft tissue lesions, and has been proved effective for restoring the impaired cartilage or preventing the aggravation [3]. However, the exact effect of PRP on the cartilage has not been compared with that of HA on animal models, especially investigated with respect to histological changes and subchondral bone restoration. In this study, P-PRP and HA were injected into the knee joint cavity of rabbits, and effects of both methods on the restoration of cartilage damage were assessed and compared.

## Materials and Methods

### The preparation of P-PRP

After the rabbits had spent one week in the cages, blood samples were extracted to prepare P-PRP according to Landesburg's protocol as reported previously [4]. Briefly, after the rabbits were anesthetized with intravenous injections of pentobarbital, 9 ml whole blood was extracted from the central auricular artery with a 10 ml syringe pre-filled with 1 ml 2.5% sodium citrate as anticoagulant. An aliquot of 20 µL of each blood sample was drawn out for cell counting, and the blood was centrifuged at 200 g for 10 min into three layers, the plasma, the platelets and the red blood cells. Then the plasma and the platelets are extracted to undergo another centrifugation at 200 g for 10 min, and most of the supernatant plasma is discarded. The plasma above the buffy coat layer was carefully collected with a pipette. The white cells were not collected in order to prepare the P-PRP. Another 20 µl of P-PRP was used for cell counting. To activate the P-PRP, 1/10 volume of CaCl_2_ was added into the liquid P-PRP, which would turn into clots with lucid serum containing abundant platelet derived growth factors. The serum was carefully collected and was then kept in −80°C for further use. To compare the concentration of the growth factors in P-PRP and in the whole blood, enzyme linked immunosorbent assay (ELISA) was performed.

### Animal models and treatment

Thirty-three adult rabbits (6–8 months old) were involved in the study. All procedures and handling to the animals was approved by the Animal Research Committee of Shanghai Jiaotong University School of Medicine. Sixty knees in thirty rabbits were used for the establishment of the cartilage defect model. Briefly, after the animals were anesthetized with intravenous injection of pentobarbital, a lateral para-patellar skin incision was made. The knee joint was exposed when the joint capsule was sliced open and the patellar was extracted laterally. In direct vision, a full-thickness cartilage and subchondral bone defect that was 5 mm in diameter and 3 mm in depth was made in the patellar groove using a 5 mm sterile stainless drill. Then the rabbits were evenly divided in a randomized manner into three groups: the P-PRP group, the HA group and the control group. And the rabbits of the three groups received 0.3 ml P-PRP, 0.3 ml low-molecular weight HA (mol wt 70,000–120,000, Sigma) solution, and 0.3 ml 0.9% saline respectively, which was injected into both knee joints once a week for 3 weeks consecutively, with the first injection administered 3 days postoperatively. The remaining 3 rabbits were used as a positive control for the micro-CT scanning and were not given surgery or injection.

### Histological assessment

Six and twelve weeks after the first injection, the rabbits were sacrificed with overdose of pentobarbital (120 mg/kg) and the bilateral distal parts of femurs were dissected. For the thirty rabbits which received surgery and injections, the samples were fixed in 4% paraformaldehyde for 48 hours, and then was dehydrated, embedded, cut and stained with H&E and Safranin-O, to observe the changes to the structure and ECM of the cartilage. The sections were examined and scored in a blind manner by three dependent trained investigators, according to Mankin's score [5] for osteoarthritis showed in table 1.

**Table 1:** Mankin's scoring for the cartilage degradation.

Items	Score
1. Structure integrity	
Intact	0
Surface irregularity	1
Irregular surface plus pannus	2
Fissures to middle zone	3
Fissures to deep zone	4
Fissures to subchondral zone	5
Totally disrupted	6
2. Chondrocytes	
Normal	0
Slight loss of chondrocytes clusters	1
<25% of the clusters	2
No clusters	3
3?Safranin-O staining	
Normal	0
Slight loss	1
Moderate	2
Slight	3
None	4
4. Tidemark	
Intact	0
Vascular penetration	1

### Micro-CT scanning

Twelve weeks after the operation, Micro-CT scanning (Skyscan1172, Bruker-microct, Belgium) was used to observe the micro-structural restoration of the subchondral bone in the defect area. After fixed in 4% formaldehyde for 48 hours, the samples were held immobile with the femoral axis perpendicular to the scanning plane. The samples were scanned through a 180°rotation angle with a rotation step of 0.6°at 18 µm resolution. The region of interest (ROI) was cylinder-shaped in this study, which was extracted from the sample with a delineation of a 5 mm circular covering the original defect area on the first slice and included 56 deeper slices (adding up to 1 mm approximately). A global threshold was used for all samples to identify bone/non-bone tissue, which was set at 68–255. Then the 3-D images were established. The quality of the regenerated bone in ROI was assessed and presented with the average bone mineral density (BMD) and bone volume/total volume (BV/TV). The samples from the 3 rabbits of the positive control were also scanned, to get the BMD and the BV/TV values calculated from the same ROI as in the other 3 groups.

### Detection of the joint fluid IL-1β level

Before the rabbits were sacrificed 12 w postoperatively, the joint cavity was flushed with 0.3 mL normal saline for 3 times, and the flushing solution from the joint cavity was collected for detection of IL-1 concentration via ELISA method.

### Statistical analysis

Data was collected from the triplicate or quadruplicate samples and were presented as mean ± standard deviation (SD). The statistical process was carried out with the SPSS 11.5 software and the statistically significant values were defined as p<0.05 or p<0.01 based on one-way analysis of variance (ANOVA).

## Results

### The assessment of the P-PRP

**Figure 1:**
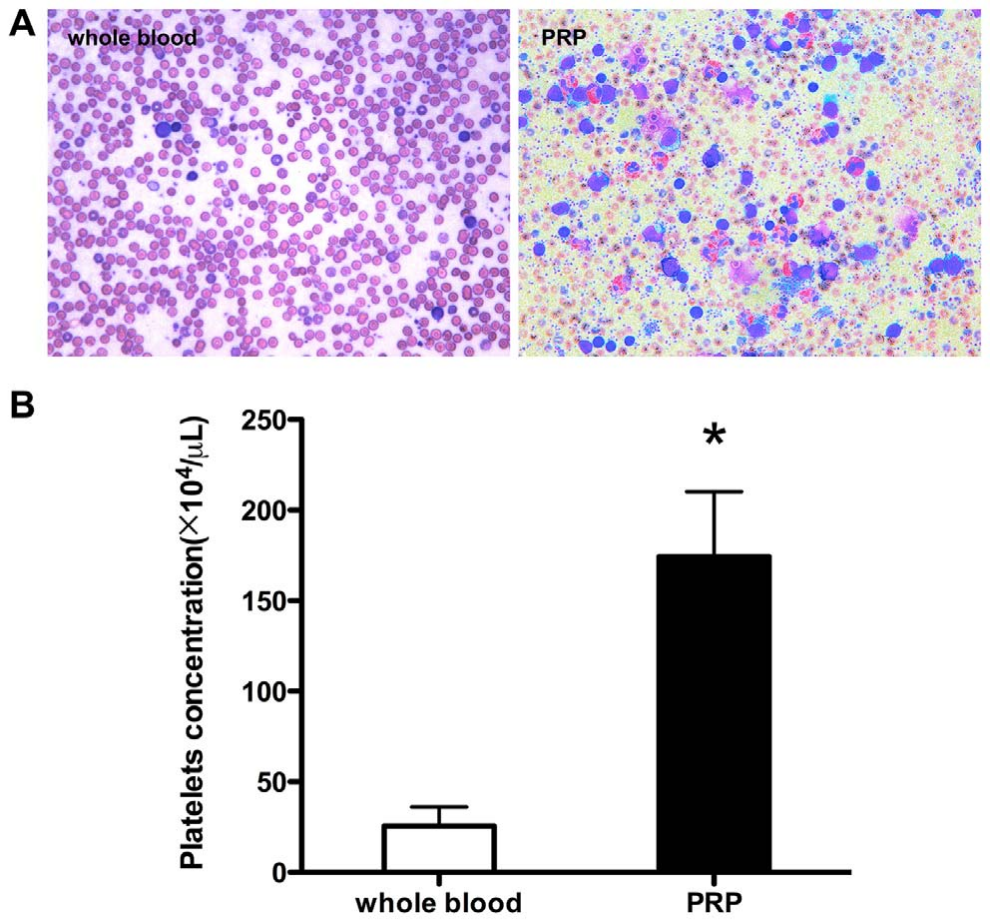
The concentrates of platelets in PRP and whole blood. (A): The smear of PRP and whole blood. Platelets and white blood cells were highly concentrated in PRP. (B): The platelets counting of PRP and whole blood. * denotes statistically different significance compared with whole blood (*p*<0.01).

The mean platelet number in the peripheral whole blood and platelet rich plasma was 25.56±10.54×10^4^/μL and 174.33±35.89×10^4^/μL, respectively. The platelet concentration in PRP is 6.8 fold of that in the whole blood (Fig. 1). And the concentrations of PDGF, TGF-β1 and bFGF are significantly higher than those in the whole blood (Table.2).

**Table 2:** Concentrations of GFs in peripheral blood and PRP.

Items	PDGF(ng/mL)	TGF-β(ng/mL)	bFGF(pg/mL)
Peripheral blood	6.78±1.72	16.21±6.6	21.04±6.12
PRP	31.22±4.3	135.19±16.8	77.34±21.3

### Gross observation

After the surgery, there was slight swelling and redness with increased skin temperature on the joints. Six weeks after the surgery, defects in the control group were filled with disrupted fibrous tissue, with little contact with surrounding cartilage, And not much changes took place but only thicker fibrous coverage could be observed after another six weeks. In the HA group, the defects didn't repair much better except the surface of the regenerative tissue was glossier. The defects treated with PRP were filled with regenerated glossy white tissue, which appeared integrated with normal tissue, though there was a slight concavity and tiny fissures in the center.

### Histological assessment

**Figure 2:**
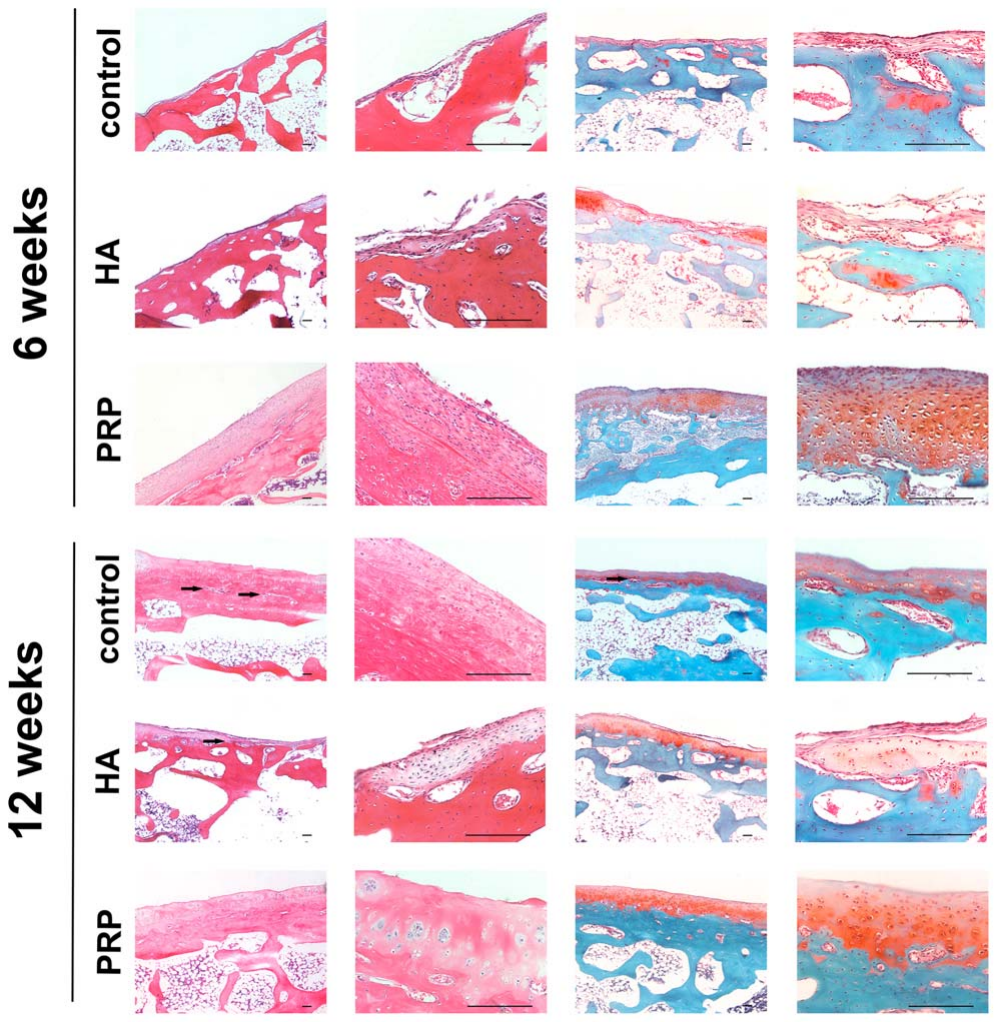
Histological assessment of cartilage restoring effects of the three groups. The left two columns were H&E stained and the right two columns with Safranin-O. For each staining, the images of the right column are magnified from the left column. The capillary infiltration areas were indicated with black arrows. All scale bars represent 200 µm.

**Figure 3:**
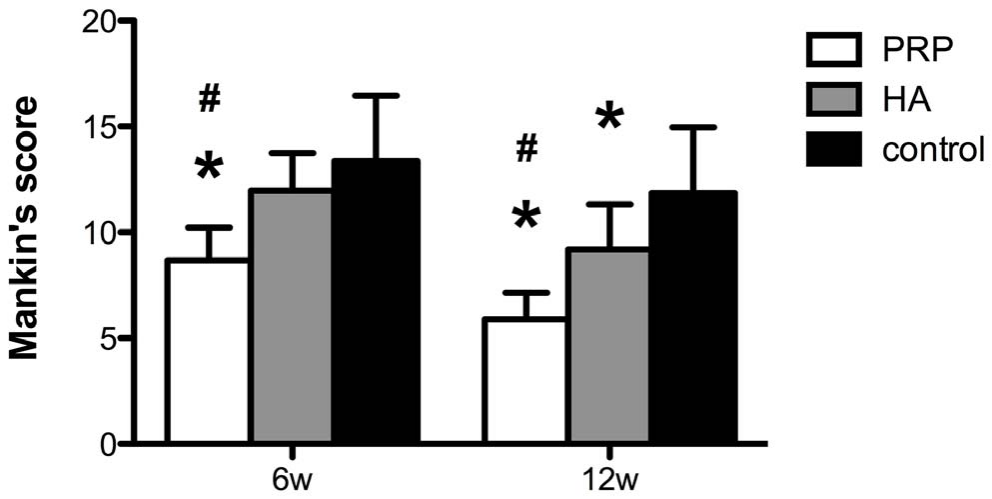
Mankin's score of the three groups at 6 w and 12 w. The score of the PRP group was lower than the other two groups at both time points. * denotes statistically significant difference compared with the control group(p<0.05). # denotes statistically significant difference compared with the HA group(p<0.05).

The regenerative tissue in the control group was thin and rough, even the subchondral bone was revealed partially with fissures. The Safranin-O staining was pale, indicating that the ECM was reduced. In the HA group, the surface of the cartilage was smoother, with a few regions of slightly restored transparent cartilaginous matrix, but the chondrocytes were still reduced and dispersed. In the PRP group, notable proliferation of chondrocytes formed in clusters can be found in the H&E stained slices, which were embedded in thick ECM as was shown in the safranin-O staining. Also capillary infiltration could be observed in the control group, which was not very obvious in the PRP group (Fig. 2). The Mankin's score of the three groups were shown in Fig. 3. The score of the PRP group was lower than the other two groups (p<0.01).

### Micro-CT assessment

**Figure 4:**
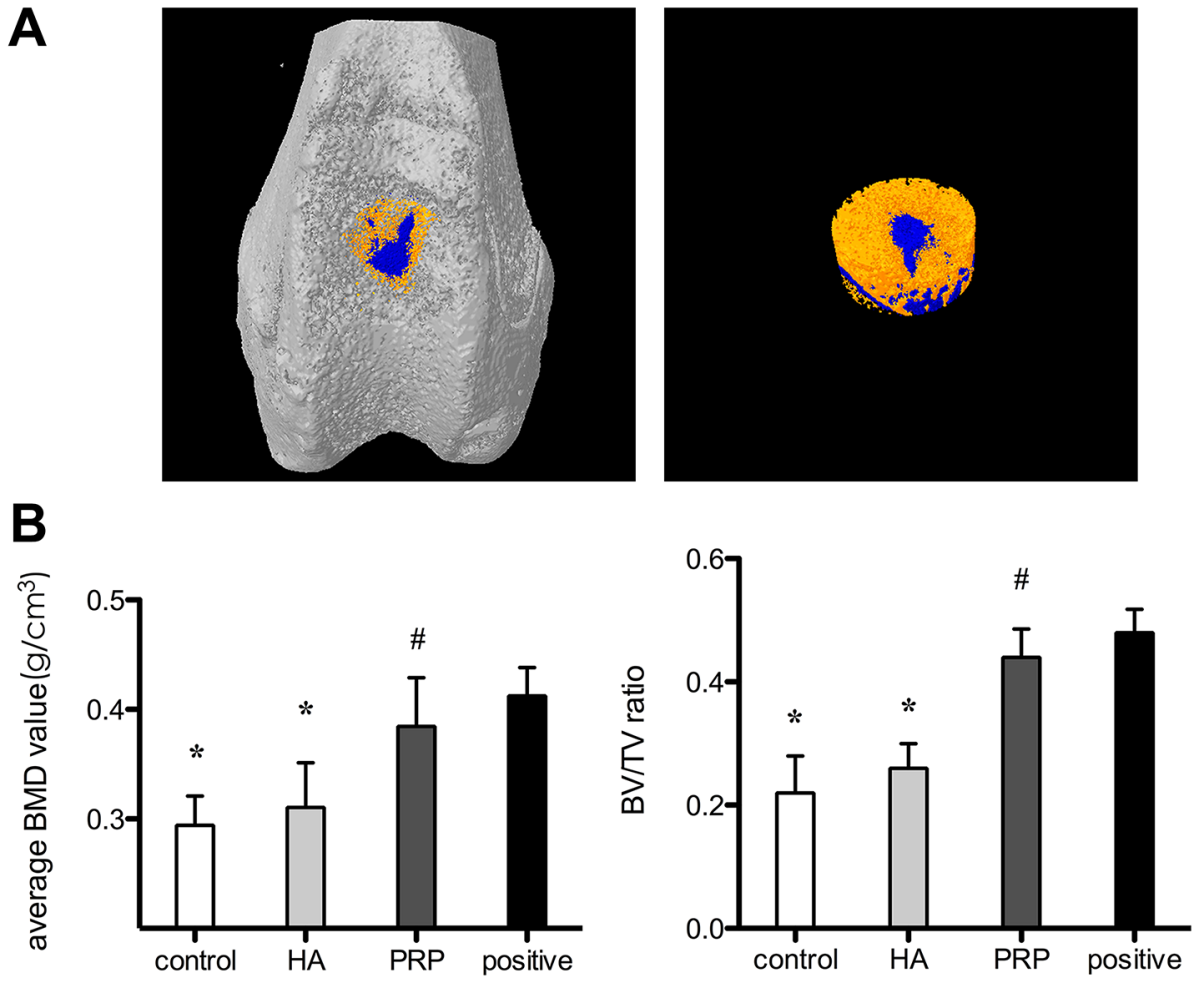
The results of micro-CT test. (A).Assessment of subchondral bone restoration in PRP group. Bright yellow represents regenerated bone and blue for tissue with lower density, such as fibrous tissue. (B).Comparison of bone mineral density(BMD) and BV/TV between the three groups and normal bone tissue(positive). *denotes statistically significant difference compared with positive group(p<0.01) and PRP group(p<0.01); #denotes none statistically significant difference compared with the positive group(p>0.05).

Micro-CT scanning and analysis was used to assess the regeneration of the subchondral bone in the defect area. The calcified regenerated subchondral bone was indicated in orange in the 3D images, and tissue with lower density such as fibrous tissue was in deep blue. The average BMD of the PRP group was the highest among the three groups (P<0.05). There was no significant difference between the average BMD values of the HA group and the control group (Fig. 4).

### The IL-1 concentration in joint fluid

**Figure 5:**
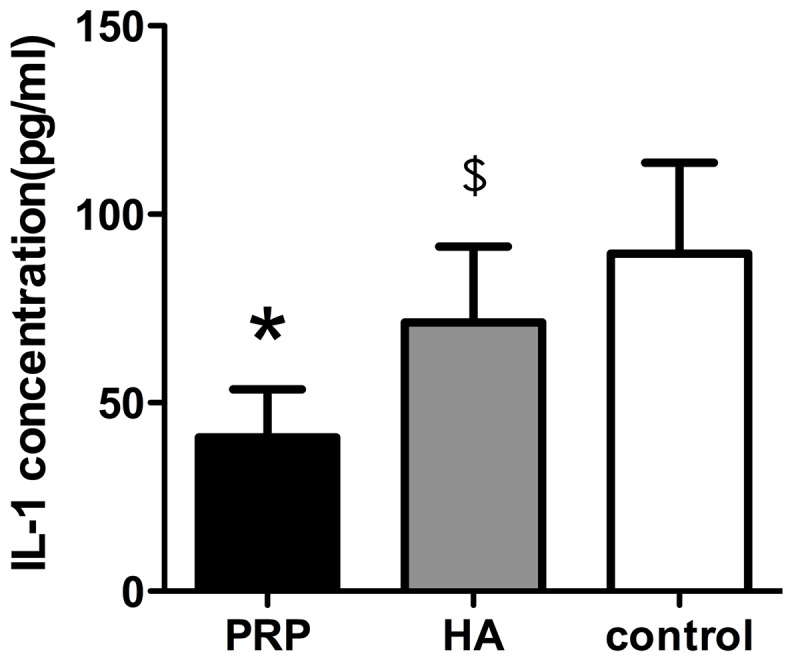
Comparison of the IL-1 concentrations in the three groups. * denotes statistically significant difference compared with HA group or the control group(p<0.01); $ denotes statistically significant difference compared with the control group(p<0.05).

The IL-1 concentration in the joint fluid was highest in the control group and lowest in the PRP group, as is shown in Fig. 5.

## Discussion

In this study, the effect of P-PRP on repairing the cartilage defect was evaluated and compared with HA. P-PRP and HA was respectively injected into rabbits' knee joints where cartilage defect model was made. The repairing effect of the two methods were compared via histological investigation, micro-CT analysis, and IL-1 test of the joint fluid. Our findings demonstrated that better histological outcomes and restoration of subchondral bone were obtained with administration of P-PRP. We also found that P-PRP could alleviate the inflammatory reaction in the joint cavity more than HA, as was indicated with the changes of IL-1 level in the joint fluid.

As mentioned above, the platelet concentrates family could be classified into four categories: P-PRP, L-PRP, P-PRF and L-PRF [2], [6], [7]. In our study, we chose the P-PRP which contains poor white cells, in that it is the restoring effect of the abundant growth factors on the cartilage defect that we intend to observe. The effects of the cell contents in PRP were not the topic of investigation of this study.

HA is a kind of acid mucopolysaccharide produced by the synovium and can lubricate the joint and protect the cartilage. In normal conditions, there is a 2 µm-thick coating of HA-protein compound on the surface of articular cartilage, conjugated with the collagen in the surface area, which can maintain the balance of the substance exchange between the joint fluid and the cartilage, and at the same time prevent the proteoglycan from being lost [8]. So this may explain why intra-articular complement of HA can relieve the degeneration of the cartilage.

The histological findings showed that an increase of chondrocytes was observed after P-PRP was injected into the joint cavity, which was demonstrated via HE staining. And it was companied by increase of production of the cartilage extracellular matrix (ECM), confirmed via safranin-O staining. It is reasonable to relate this effect to the high concentration of the growth factors released from P-PRP, which were produced by activated platelets and can promote the restoration of the injured tissues [9]. Moreover, the relation and interaction between the factors is the key point of the P-PRP potency, and can work far more efficiently than one single component. Also, it's been reported that P-PRP can stimulate the articular chondrocyte proliferation and matrix biosynthesis [3]. Among the growth factors from the platelets, IGF can stimulate the chondrocyte proliferation as well as the matrix synthesis [10]; TGF-β can also induce the transformation of the chondrocytes and the deposit of the collagen [11]. These bioactive growth factors accounted for the superiority of P-PRP over HA.

Besides, the micro-CT scanning showed the reconstructive effect of P-PRP on the subchondral bone, which was not found in the HA group or the control group. The subchondral area is quite crucial in cartilage reconstruction as it supports the overlying neocartilage tissue. In our study, the tests of BMD and BV/TV were run concerning the originally defected area in all the groups. These results indicated both the quantity and the quality of the neo-subchondral bone, which were highest in the P-PRP group. This proved that P-PRP could effectively promote the maturation as well as the calcium depositing of the subchondral bone. While in the HA group or the control group, less bone mass was formed. This difference could also be attributed to the growth factors in P-PRP. There has been studies demonstrating beneficial effects of P-PRP on bone defect healing and bone formation, both experimentally [12]–[14] and clinically [15], [16].

Moreover, it was also verified in this experiment that P-PRP showed explicit alleviation on the osteoarthritic changes, as was supported by the detection of IL-1β concentration in the joint fluid. Inflammatory factors such as IL-1β are indicators of the existence and severity of osteoarthritis. And IL-1β will promote the production of NO and accelerate the degradation of the cartilage by up-regulating the matrix metalloproteinase (MMP-1,13) and down-regulating the synthesis of the ECM [17]. So the concentration of IL-1β in the synovial fluid and in the serum would be obviously up-regulated, while there is barely any in normal joints.

The anti-osteoarthritic effect of P-PRP may be the result from the relief of the cartilage damage, which at the same time reduced the local inflammation as well as the stimulation to the synovium, causing a drop in the IL-1β secretion. On the other hand, P-PRP has a direct influence on the synovium, as it can lower the NF-κB activity, and suppress the expression of COX-2 and CXCR4, which is the important regulatory factor in the inflammatory reactions. Meanwhile, PRP can up-regulate the expression of HGF, IL-4 and TNF-α, while HGF and TNF-α can block the expression of NF-κB to inhibit the inflammation [18]. All these bio-active features account for the superiority of PRP over HA, which acts as lubricant between the fricative articular surfaces and complement to the cartilage ECM, but no efficacy to the restoration of lost chondrocytes.

As one of the novel biological treatments for cartilage lesions, PRP has been exploited widely in recent years, especially in clinical application [19]–[21]. In 2010, Kon et al. [22] published a prospective study on 115 knees of 91 patients, which were treated with 3 injections of 5 mL PRP (1 every 3 weeks). The satisfactory rate was eighty percent within the first 6 months, whereas a tendency to worsen was reported at 12 months of follow-up. The authors confirmed the time-dependency of intra-articular therapy with platelet-derived GFs and estimated the median duration of the PRP effect to be 9 months. Another multi-center study [23] compared the efficacy of PRP and HA intra-articular injections for the treatment of knee cartilage degenerative lesions and osteoarthritis, which revealed that better results were achieved in younger and more active patients with a low degree of cartilage degeneration. And this finding agreed with the results of our experiments. The cartilage defect, or a fresh wound to the cartilage was treated with PRP within one week, which was actually an early stage of cartilage lesion, and revealed better response to PRP than HA.

## Conclusion

From the results of the study, it can be drawn that P-PRP can effectively restore the defected cartilage, and alleviate the inflammatory reaction of the joint. Moreover, P-PRP is prepared totally from the autologous whole blood and is therefore safe and of low cost, enabling it as a possible substitute to HA in treating degeneration or damage of articular cartilage.
